# Associations between chronic comorbidity and exacerbation risk in primary care patients with COPD

**DOI:** 10.1186/s12931-017-0512-2

**Published:** 2017-02-06

**Authors:** Janine A. M. Westerik, Esther I. Metting, Job F. M. van Boven, Waling Tiersma, Janwillem W. H. Kocks, Tjard R. Schermer

**Affiliations:** 1 Department of Primary and Community Care, Radboud University Medical Center, 117-ELG, Geert Grooteplein Noord 21, Nijmegen, 6525 EZ The Netherlands; 2 Department of General Practice, Groningen Research Institute for Asthma and COPD (GRIAC), University Medical Center Groningen, University of Groningen, HPC FA21, Antonius Deusinglaan 1, Groningen, 9713 AV The Netherlands

## Abstract

**Background:**

COPD often coexists with chronic conditions that may influence disease prognosis. We investigated associations between chronic (co)morbidities and exacerbations in primary care COPD patients.

**Method:**

Retrospective cohort study based on 2012–2013 electronic health records from 179 Dutch general practices. Comorbidities from patients with physician-diagnosed COPD were categorized according to International Classification of Primary Care (ICPC) codes. Chi-squared tests, uni- and multivariable logistic, and Cox regression analyses were used to study associations with exacerbations, defined as oral corticosteroid prescriptions.

**Results:**

Fourteen thousand six hundred three patients with COPD could be studied (mean age 67 (SD 12) years, 53% male) for two years. At baseline 12,826 (88%) suffered from ≥1 comorbidities, 3263 (22%) from ≥5. The most prevalent comorbidities were hypertension (35%), coronary heart disease (19%), and osteoarthritis (18%). Several comorbidities showed statistically significant associations with frequent (i.e., ≥2/year) exacerbations: heart failure (odds ratio [OR], 95% confidence interval: 1.72; 1.38–2.14), blindness & low vision (OR 1.46; 1.21–1.75), pulmonary cancer (OR 1.85; 1.28–2.67), depression 1.48; 1.14–1.91), prostate disorders (OR 1.50; 1.13–1.98), asthma (OR 1.36; 1.11–1.70), osteoporosis (OR 1.41; 1.11–1.80), diabetes (OR 0.80; 0.66–0.97), dyspepsia (OR 1.25; 1.03–1.50), and peripheral vascular disease (OR 1.20; 1.00–1.45). From all comorbidity categories, having another chronic respiratory disease beside COPD showed the highest risk for developing a new exacerbation (Cox hazard ratio 1.26; 1.17–1.36).

**Conclusion:**

Chronic comorbidities are highly prevalent in primary care COPD patients. Several chronic comorbidities were associated with having frequent exacerbations and increased exacerbation risk.

## Background

Although nowadays healthcare systems are largely configured to manage individual diseases rather than multimorbidity, there is an increasing awareness of the importance of comorbidities in patients with chronic conditions [1]. Chronic obstructive pulmonary disease (COPD), a prevalent chronic respiratory condition, is a major cause of morbidity and mortality worldwide [2]. In the past decade several studies have shown that COPD often coexists with other diseases, [3, 4] and that comorbidity is associated with poorer clinical outcomes [4, 5]. Some of these comorbidities arise independently of COPD, whereas others may be causally related, either through shared risk factors (smoking, aging) or shared pathophysiology, as a complication of COPD, or due to medication side effects.

Several associations between COPD and particular comorbidities have been shown. Cardiovascular disease, metabolic syndrome, skeletal muscle dysfunction, osteoporosis, depression and lung cancer are all highly prevalent among patients with any severity of COPD, and cross-sectional studies have shown their significant impact on patients’ health-related quality of life [2, 6, 7]. Most of the research on comorbidity in COPD comes from studies in secondary care populations, thus representing patients in the more severe part of the COPD severity spectrum [4]. However, in most developed countries, the vast majority of patients with COPD are managed in primary care. Studies performed in general practice settings report that 21 to 74% of patients with COPD suffer from two or more additional chronic diseases [6, 8].

As COPD is a progressive disease, factors that influence its prognosis are important to consider when managing patients. Since exacerbation frequency is a known predictor of COPD progression, [2] it is important to know what the potential impact of comorbidities on the risk of exacerbations is. Recently Putcha et al*.*reported a model in which the number of comorbid conditions predicted dyspnea and exacerbation risk [9]. This prediction model does, however, not take into account which particular comorbid conditions are associated with exacerbation risk. Other previous studies have predominantly looked at mortality as the outcome of interest, [5, 10, 11] but from a patient management perspective it is important that physicians consider comorbidities that influence potentially modifiable prognostic factors like exacerbation rate in their treatment decisions. Therefore, the aim of the current study was to explore associations between a wide range of comorbid chronic conditions and exacerbation risk in a real-life cohort of primary care patients with COPD.

## Methods

### Design and dataset

The study used routine data from a general practice database from the Department of Primary and Community Care at the Radboud University Medical Center, Nijmegen, the Netherlands. De-identified electronic medical records from primary care patients diagnosed with COPD from 179 general practices in the eastern part of the Netherlands were available in the database.

For each registered subject, the following data were extracted: age, sex, all diagnoses using the International Classification of Primary Care (ICPC), extended with Dutch ICPC sub-codes, [12] and all prescribed medication. ICPC-2 or ICD10 coding data were recoded into ICPC-1. Medication prescriptions (i.e., prescription start and end dates, dosage, frequency, and duration) were extracted and categorized using the Anatomical Therapeutic Chemical (ATC) classification system [13]. For the current study only the data on prescriptions for oral corticosteroids were used.

### Study population

Subjects aged ≥40 years were included in the study population when they had physician-diagnosed COPD (as labeled with ICPC code R95 in the electronic medical record) before or during the study period. Asthma (ICPC R96) in addition to the COPD code was not an exclusion criterion. The follow-up period covered the years 2012 and 2013. The observation period for patients terminated either at the end of the study period (31 December 2013), or when a subject died or deregistered from the practice.

### Comorbidities

The selection of chronic comorbid diseases studied was based on existing literature [1, 14], the authors’ clinical expertise and expert opinions (Nielen MM, Spronk I, Davids R, Korevaar JC, Poos MJ, Hoeymans N, Opstelten W, van der Sande MAB, Biermans MCJ, Schellevis FG, RA V: A new method for estimating morbidity rates based on routine electronic medical records in primary care, submitted). We considered all chronic diseases as comorbidities, regardless whether the disease had been diagnosed before the COPD diagnosis or thereafter. Apart from all ‘obligatory’ chronic diseases we also included several recurrent diseases (i.e., depression, anxiety, anemia, dyspepsia, urinary tract infection) which could potentially influence COPD outcomes. After reaching consensus about these recurrent comorbidities within the research team, ICPC (sub)codes were linked (see Appendix 1). Selection of the recurrent comorbidities in our population was based on the patient’s history in terms of these particular ICPC codes. To define whether a history of ICPC codes was relevant or irrelevant for the aim of the study, we added specific selection criteria based on published clinical guidelines for the respective diseases (see Appendix 1).

Finally, a total of 82 chronic comorbid conditions were selected and included in the analyses. The comorbidities were clustered and analyzed based on their ICPC codes into the following 14 categories: respiratory; cardiovascular; digestive; endocrine; metabolic/nutrition; musculoskeletal; neurologic; psychiatric; urogenital; blood (−forming organs)/lymphatics; infectious; eye/ear/skin; non-pulmonary cancer; and pulmonary cancer. Low prevalence categories were merged (see Appendix 2). To restrict ourselves, we focused on conditions with a high prevalence and cardiopulmonary comorbidities (other than COPD) with a lower prevalence (7 conditions, see Table 2). High-prevalent comorbidities (19 conditions), further referred to as ‘frequent comorbidities’, were defined as being present in ≥5% of the study population. This resulted in a total of 26 comorbidities remaining for further analyses.

### Outcomes

The outcomes for the study were (i) prevalence of comorbidities in the study population, (ii) annual rate of exacerbations (dichotomized as <2 *versus* ≥2 exacerbations/year based on the cumulated 2012/13 data), and (iii) time (in days) until first exacerbation. An exacerbation was defined as a prescription of oral corticosteroids (i.e., prednisolone (ATC H02AB06) or prednisone (ATC H02AB07)) with a minimum daily dose of 20 mg for a minimum duration of 5 days and a maximum duration of 15 days (based on Dutch GP guidelines for treatment of COPD exacerbations [15]). As there is no consensus in the literature regarding a cut-off to differentiate between relapse of an earlier exacerbation and a new exacerbation, [16] we considered a subsequent predniso(lo)ne prescription after an oral corticosteroid-free interval of ≥14 days since the end-date of the previous prescription as a new exacerbation.

### Statistical analysis

Analyses were performed with SPSS statistical software (version 22, IBM SPSS Statistics, Feltham, Middlesex, UK) and Microsoft Excel 2007 (Microsoft Corporation, Redmond, Washington, US). Statistically significant results were defined as *p* < 0 · 05. Patients’ baseline characteristics and comorbidity prevalence rates were calculated. We performed Chi-square tests for categorized variables and independent t-tests for continuous variables to analyze differences between the subgroups with <2 and ≥2 exacerbations per year.

We explored associations between comorbidities and exacerbation risk using univariable analyses. Hazard ratios for comorbidities were calculated using Cox regression, in which the time variable consisted of time to the first exacerbation. Data from patients who died or were otherwise lost to follow up were right-censored. Subsequently, all frequent and cardiopulmonary comorbidities (Table 2), age, and gender were included as covariates in multivariate Cox regression analyses. The model was reduced through backward exclusion to produce a final model that consisted of only non-collinear, independently associated, statistically significant covariates. The same modeling approach was used for comorbidity categories using all other categories, with age and gender as covariates.

In addition, we performed multivariable logistic regression analyses to calculate odds ratio’s (ORs) with the dichotomous indicator variable for exacerbation frequency (<2 *versus* ≥2 exacerbations/year) as the dependent variable. Predictor variables in the logistic models were: all frequent comorbidities, all cardiopulmonary comorbidities, gender, and age. This modeling approach was also used to analyze the 14 categories of comorbidity.

## Results

### Study population

Overall, data of 16,427 subjects diagnosed with COPD were available for analyses. Of these patients, 1824 (11 · 1%) were lost to follow-up during the 2-year study period. Reason for loss to follow-up was known for 800 (44 · 5%) of these patients, with death being the predominant reason. Table 1 shows baseline characteristics of the patients with complete follow-up (i.e., the final study population, *n* = 14,603). Mean (SD) age was 66 · 5 (11 · 5) years and 53% were males. At baseline, 89 · 1% of patients suffered from ≥1 chronic comorbid conditions, while 23 · 1% had ≥5 comorbidities. Most prevalent comorbid conditions were hypertension (35 · 2%), coronary heart disease (19 · 2%), osteoarthritis (17 · 6%), diabetes (17 · 3%), and peripheral vascular disease (14 · 3%). Table 2 shows the prevalence rates of the frequent and cardiopulmonary comorbidities. Table 3 shows the prevalence of ICPC-categorized comorbidities.

**Table 1:** Baseline characteristics of the COPD study population grouped by low (<2/year) *versus* high (≥2/year) exacerbation rate

	Patients with full follow-up (study population)^a^ (*n* = 14,603)	Subgroups of study population	
Patient characteristics		Patients with <2 exacerbations/year (*n* = 13,709)	Patients with ≥2 exacerbations/year (*n* = 894)^b^
Sex, male, n (%)	7,749 (53 · 1)	7,322 (53 · 4)	427 (47 · 8)^‡^
Age at study baseline, years; mean (SD; range)	66 · 5 (11 · 5; 40–110)^‡^	66 · 5 (11 · 6; 40–110)	67 · 4 (10 · 3; 40–93)^‡^
Full dataset available (censored data), n (%)			
Full data available		13,709 (93 · 9)	894 (6 · 1)
Deceased	N/A	N/A	N/A
Moved	N/A	N/A	N/A
Nursing home	N/A	N/A	N/A
Unknown	N/A	N/A	N/A
Comorbidity data			
Number of comorbid diseases^c^, mean (SD; range)	3 · 0 (2 · 3;0–20)^‡^	3 · 0 (2 · 3;0–16)	3 · 4 (2 · 5; 0–20)^‡^
Number of comorbid diseases categories^c^, n (%)			
0	1,777 (12 · 2)	1,700 (12 · 4)	77 (8 · 6)
1 or 2	5,305 (36 · 6)	5,021 (36 · 6)	284 (31 · 8)
3 or 4	4,258 (29 · 2)	3,977 (29 · 0)	281 (31 · 4)
5 and more	3,263 (22 · 3)^‡^	3,011 (22 · 0)	252 (28 · 2)^‡^
Exacerbations			
Number of exacerbations^d^, mean (SD; range)	0 · 75 (1 · 5;0–15)^‡^	0 · 44 (0 · 8;0–2)	5 · 6 (2 · 0;3–15)^‡^

*SD* standard deviation, *N/A* not applicable

^*^ *p* < 0.05, ^†^ *p* < 0.01, ^‡^ *p* < 0.001

^a^ *p*-values displayed are calculated for the difference between patients lost to follow-up *versus* patients with full follow-up. Chi-square tests for categorized variables and independent t-tests for continuous variables. *p* < 0 · 05 was considered statistically significant

^b^ *p*-values displayed are calculated for the difference between the subgroups <2 *versus* ≥2 exacerbations/year. Chi-square tests for categorized variables and independent t-tests for continuous variables. *p* < 0 · 05 was considered statistically significant

^c^presence of any type of comorbid disease was assessed at study baseline, i.e., 1 January 2012

^d^Mean number of exacerbations during the study period, 1 January 2012 – 31 December 2013

Baseline characteristics of the initial population of all COPD patients (*n* = 16,427) and those who were lost to follow-up (*n* = 1,824) are reported in Appendix 3

**Table 2:** Prevalence of frequent and cardiopulmonary comorbidity in the study population, sorted from highest to lowest prevalence rate

	Total study population^a^, (*n* = 14,603)	Patients with <2 exacerbations/year, (*n* = 13,709)	Patients with ≥2 exacerbations/year, (*n* = 894)	*p*-value^b^
Frequent comorbidity				
Hypertension	5,116 (35 · 0)	4,805 (35 · 2)	311 (34 · 8)	0 · 873
Coronary heart disease	2,759 (18 · 9)	2,569 (18 · 7)	191 (21 · 4)	0 · 051
Osteoarthritis	2,570 (17 · 6)	2,402 (17 · 5)	168 (18 · 8)	0 · 334
Diabetes	2,464 (16 · 9)	2,330 17 · 0)	134 (15 · 0)	0 · 120
Peripheral vascular disease	2,031 (13 · 9)	1,897 (14 · 8)	150 (16 · 8)	*0 · 006*
Blindness & low vision	1,938 (13 · 3)	1,772 (12 · 9)	166 (18 · 6)	*<0 · 001*
Dyspepsia, gastroesophageal reflux	1,845 (12 · 6)	1,703 (12 · 4)	142 (15 · 9)	*0 · 003*
Dislipidemia	1,703 (11 · 7)	1,613 (11 · 8)	90 (10 · 1)	0 · 125
Stroke & transient ischaemic attack	1,357 (9 · 3)	1,259 (9 · 2)	98 (11 · 0)	0 · 076
Chronic kidney diease	1,360 (9 · 3)	1,263 (9 · 2)	97 (10 · 9)	0 · 103
Asthma	1,305 (8 · 9)	1,202 (8 · 8)	103 (11 · 5)	*0 · 005*
Hearing loss	1,144 (7 · 8)	1,078 (7 · 9)	66 (7 · 4)	0 · 604
Heart failure	1,048 (7 · 2)	943 (6 · 9)	105 (11 · 7)	*<0 · 001*
Atrial fibrillation	1,044 (7 · 1)	964 (7 · 0)	80 (8 · 9)	0 · 031
Skin cancer	913 (6 · 3)	862 (6 · 3)	51 (5 · 7)	0 · 485
Osteoporosis/osteopenia	884 (6 · 1)	801 (5 · 8)	83 (9 · 3)	*<0 · 001*
Thyroid disorder	808 (5 · 5)	757 (5 · 5)	51 (5 · 9)	0 · 817
Depression	800 (5 · 5)	729 (5 · 3)	71 (7 · 9)	*0 · 001*
Prostate disorders	784 (5 · 4)	719 (5 · 2)	65 (7 · 3)	*0 · 009*
Cardiopulmonary comorbidity				
Heart valve disease	568 (3 · 9)	528 (3 · 9)	40 (7 · 8)	*0 · 035*
Bronchiectasis/chronic bronchitis	414 (2 · 8)	379 (2 · 8)	35 (3 · 9)	0 · 045
Pulmonary cancer	317 (2 · 2)	284 (2 · 1)	33 (3 · 7)	*0 · 001*
Sleep apneu syndrome	173 (1 · 2)	161 (1 · 2)	12 (1 · 3)	0 · 653
Other chronic pulmonary disease	157 (1 · 1)	148 (1 · 1)	9 (1 · 0)	0 · 838
Recurrent sinusitis	54 (0 · 4)	49 (0 · 4)	55 (6 · 2)	0 · 335
Congenital cardiovascular anomaly	32 (0 · 2)	28 (0 · 2)	4 (0 · 4)	0 · 132

^a^COPD population with complete data available, patients lost to follow-up (*n* = 1,824) excluded

^b^ *p*-values displayed are calculated for the difference between the subgroup <2 *versus* ≥2 exacerbations/year Chi-square tests for categorized variables. *p* < 0 · 05 was considered statistically significant

**Table 3:** Prevalence of ICPC-categorized comorbidity in the COPD study population, sorted from highest to lowest prevalence rate of frequent exacerbations

	Study population^a^, (*n* = 14,603)	Patients with <2 exacerbations/year, (*n* = 13,709)	Patients with ≥2 exacerbations/year (*n* = 894)	*p*-value^b^
Comorbidity category				
Cardiovascular	8,516 (58 · 3)	7,955 (58 · 0)	561 (62 · 8)	0 · 006
Endocrine, metabolic and nutrition	4,856 (33 · 3)	4,568 (33 · 3)	288 (25 · 5)	0 · 496
Musculoskeletal	3,588 (24 · 6)	3,337 (24 · 3)	251 (28 · 1)	0 · 012
Eye and ear	2,984 (20 · 4)	2,762 (20 · 1)	222 (24 · 8)	0 · 001
Digestive	2,801 (19 · 2)	2,597 (18 · 9)	204 (22 · 8)	0 · 004
Urogenital (male and female)	2,330 (16 · 0)	2,146 (15 · 7)	184 (20 · 6)	<0 · 001
Psychiatric	2,271 (15 · 6)	2,092 (15 · 3)	179 (20 · 0)	<0 · 001
Non-pulmonary cancer	2,203 (15 · 1)	2,071 (15 · 1)	132 (14 · 8)	0 · 782
Respiratory (excl · pulmonary cancer)	1,998 (13 · 7)	1,839 (13 · 4)	159 (17 · 8)	<0 · 001
Skin	1,395 (9 · 6)	1,314 (9 · 6)	81 (9 · 1)	0 · 605
Neurological	413 (2 · 8)	389 (2 · 8)	24 (2 · 7)	0 · 789
Pulmonary cancer	317 (2 · 2)	284 (2 · 1)	33 (3 · 7)	0 · 001
Blood (forming organs) and lymphatics	106 (0 · 7)	97 (0 · 7)	9 (1 · 0)	0 · 307
Infectious	87 (0 · 6)	80 (0 · 6)	7 (0 · 8)	0 · 453

*ICPC* International Classification of Primary Care

^a^Total COPD population, with patients who were lost to follow-up (*n* = 1,824) excluded

^b^ *p*-values displayed are calculated for the difference between the group <2 *versus* ≥2 exacerbations/year. We performed Chi-square tests for categorized variables. *p*-value <0 · 05 was considered statistically significant

During the 2-year study period the mean number of exacerbations per patient was 0.72 (SD 1 · 5). 68% of patients had no exacerbation and 5 · 7% had ≥4 exacerbations during the study period.

### Associations between comorbidities and exacerbation frequency

Tables 2 and 3 show the univariable associations between comorbidities and comorbidity categories and the exacerbation frequency subgroups, respectively. Overall, patients with one or more comorbid conditions more often had ≥2 exacerbations/year compared to patients without any comorbidity (5 · 9% *vs* 4 · 0%, *p* = 0 · 001). Patients with any other chronic respiratory disease next to their COPD, (*n* = 2,294, 15 · 7%) more often had ≥2 exacerbations per year compared to patients without respiratory comorbidity (8 · 2% vs 5 · 7%, *p* < 0 · 001).

**Fig. 1:**
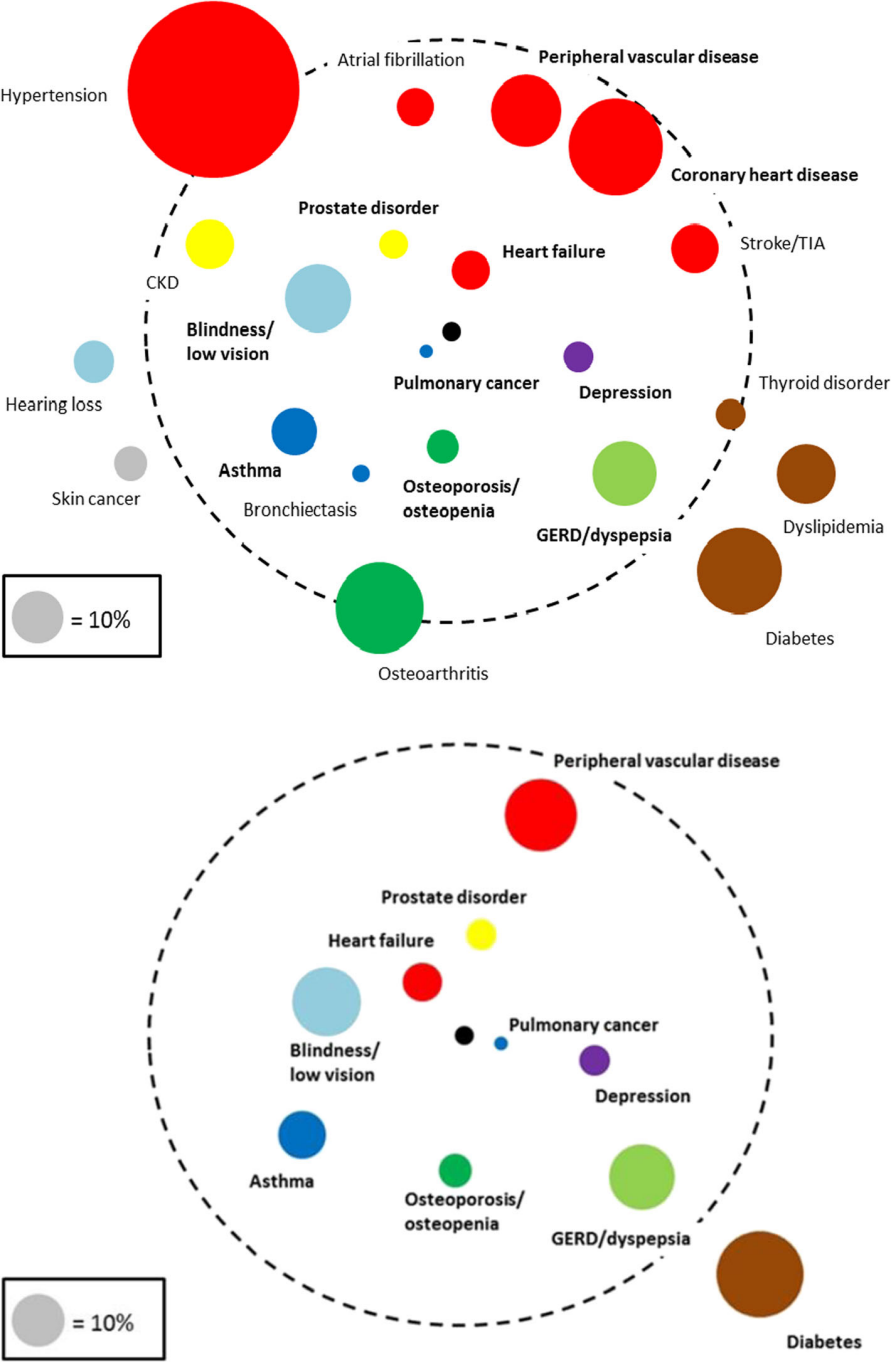
Comorbidome of comorbidities in the COPD study population (*n* = 14,603). Results are from univariable (upper panel) and multivariable (lower panel, corrected for age, gender and the other comorbidities) logistic regression analysis. (Diameter of the coloured circles represents the prevalence of each comorbidity. Proximity to the black centre of the circle represents stronger positive association (OR) with ≥2 exacerbation per year. The dashed circle represents an OR of 1. Comorbidities marked **bold** were statistically significantly (i.e., *p* < 0.05) associated with increased or decreased risk. In the multivariable model covariates were sequentially dropped until only statistically significant covariates remained. Comorbidities outside the dashed circle were negatively associated (i.e., ‘protective’) with ≥2 exacerbation/year. Comorbidities with prevalence <5% were not analysed). CKD: chronic kidney disease. COPD: chronic obstructive pulmonary disease. GERD: gastroesophageal reflux disease. TIA: transient ischemic attack

Univariable logistic regression analysis showed that COPD patients with pulmonary cancer had 1.81 higher odds for ≥2 exacerbations per year compared to patients without pulmonary cancer (Fig. 1, *p* = 0.002). Patients who, next to their COPD, also suffered from asthma, blindness or low vision, coronary heart disease, depression, dyspepsia, heart failure, osteoporosis or osteopenia, peripheral vascular disease, or prostate disorders, had a higher risk of having frequent exacerbations compared to those who did not suffer from these comorbid conditions (Fig. 1).

Table 4 lists the comorbidities and comorbidity categories significantly associated with having ≥2 exacerbation per year. In the multivariable logistic regression analysis, among the statistically significant associations, the highest ORs for having ≥2 exacerbations per year were observed for pulmonary cancer (OR 1 · 85; 95% CI 1 · 28–2 · 67), heart failure (OR 1 · 72; 1 · 38–2 · 14), prostate disorders (OR 1 · 50; 1 · 13–1 · 98) and blindness/low vision (OR 1 · 46; 1 · 21–1 · 75) as comorbid conditions (Table 4). Dislipidemia was not statistically significant, but did show a trend, with an OR of 0 · 81 (95% CI 0 · 65–1 · 01, *p* = 0 · 071). When looking at comorbidity categories, patients with other chronic respiratory conditions (OR 1 · 37; 1 · 15–1 · 64) and psychiatric comorbidities (OR 1 · 35; 1 · 13–1 · 60) were at highest risk for frequent exacerbations.

**Table 4:** Comorbidities associated with ≥2 exacerbations/year versus <2 exacerbations/year in COPD patients, corrected for age and sex (multivariable results), sorted by *p*-value

	Odds ratio (95%CI)	*p*-value
Comorbid conditions^a, b^		
Heart failure	1 · 72 (1 · 38–2 · 14)	<0 · 001
Blindness & low vision	1 · 46 (1 · 21–1 · 75)	<0 · 001
Pulmonary cancer	1 · 85 (1 · 28–2 · 67)	0 · 002
Depression	1 · 48 (1 · 14–1 · 91)	0 · 003
Prostate disorders	1 · 50 (1 · 13–1 · 98)	0 · 004
Asthma	1 · 36 (1 · 11–1 · 70)	0 · 004
Osteoporosis/osteopenia	1 · 41 (1 · 11–1 · 80)	0 · 006
Diabetes	0 · 80 (0 · 66–0 · 97)	0 · 020
Dyspepsia, gastroesophageal reflux	1 · 25 (1 · 03–1 · 50)	0 · 023
Peripheral vascular disease	1 · 20 (1 · 00–1 · 45)	0 · 049
Comorbidity categories^b,c^		
Respiratory (excl. pulmonary cancer)	1 · 37 (1 · 15–1 · 64)	<0 · 001
Psychiatric	1 · 35 (1 · 13–1 · 60)	<0 · 001
Urogenital (male and female)	1 · 34 (1 · 12–1 · 60)	<0 · 001
Eye and ear	1 · 25 (1 · 06–1 · 47)	0 · 007
Endocrine, metabolic and feeding	0 · 85 (0 · 73–0 · 99)	0 · 032
Cardiovascular	1 · 17 (1 · 01–1 · 36)	0 · 037

*OR* odds ratio

^a^All chronic comorbidities with prevalence ≥5% and cardiopulmonary comorbidities were included in the multivariable logistic regression model

^b^Reference category was ‘comorbidity not diagnosed before study period’ (i.e., 1 January 2012)

^c^All ICPC comorbidity categories were included in the multivariate logistic regression mode

### Time to first exacerbation

Table 5 summarizes the results from the Cox regression analyses. Among the statistically significant associations, the comorbid conditions with the highest risk of developing a first exacerbation were recurrent sinusitis (Cox hazard ratio 1 · 53; 95% CI, 1 · 05–2 · 24), bronchiectasis/chronic bronchitis (HR = 1.50; 1.31–1.73) and heart failure (1 · 41; 1 · 29–1 · 55). For dislipidemia a non-statistically HR of 0 · 92 was observed (*p* = 0 · 067, 95% CI 0 · 85–1 · 00).

**Table 5:** Comorbidities associated with development of a first exacerbation in the study population, corrected for age and sex (results from multivariable Cox regression analysis), sorted by *p*-value

	Cox hazard ratio (95% CI)	*p*-value
Comorbidity^a,b^		
Bronchiectasis/chronic bronchitis	1 · 50 (1 · 31–1 · 73)	<0 · 001
Heart failure	1 · 41 (1 · 29–1 · 55)	<0 · 001
Depression	1 · 34 (1 · 20–1 · 50)	<0 · 001
Atrial fibrillation	1 · 27 (1 · 16–1 · 40)	<0 · 001
Asthma	1 · 24 (1 · 14–1 · 36)	<0 · 001
Peripheral vascular disease	1 · 15 (1 · 07–1 · 24)	<0 · 001
Prostate disorders	1 · 20 (1 · 04–1 · 45)	0 · 002
Blindness & low vision	1 · 11 (1 · 03–1 · 20)	0 · 009
Coronary heart disease	1 · 10 (1 · 02–1 · 17)	0 · 011
Dyspepsia, gastroesophageal reflux	1 · 10 (1 · 02–1 · 20)	0 · 013
Pulmonary cancer	1 · 23 (1 · 04–1 · 45)	0 · 016
Recurrent sinusitis	1 · 53 (1 · 05–2 · 24)	0 · 028
Osteoporosis/osteopenia	1 · 12 (1 · 01–1 · 25)	0 · 037
Comorbidity category^b, c^		
Respiratory (excl. pulmonary cancer)	1 · 26 (1 · 17–1 · 36)	<0 · 001
Urogenital (male and female)	1 · 18 (1 · 10–1 · 27)	<0 · 001
Cardiovascular	1 · 16 (1 · 08–1 · 24)	<0 · 001
Mental health	1 · 16 (1 · 08–1 · 24)	<0 · 001
Eye and ear	1 · 09 (1 · 02–1 · 16)	0 · 013
Digestive	1 · 07 (1 · 00–1 · 15)	0 · 042

^a^All chronic comorbidities with prevalence ≥5% and cardiopulmonary comorbidities were included in the multivariate Cox regression model

^b^Reference category was ‘comorbidity not diagnosed before study period’ (i.e., 1 January, 2012)

^c^All ICPC comorbidity categories were included in the multivariate Cox regression model

**Fig. 2:**
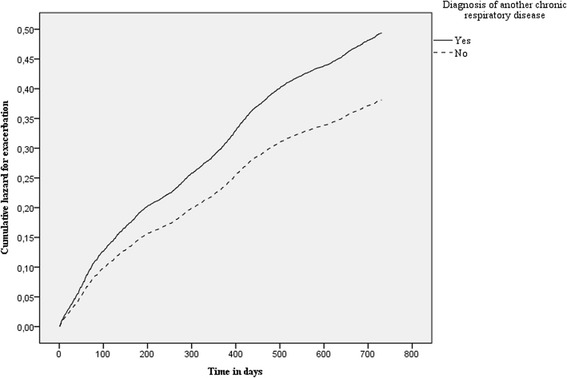
Hazard for exacerbation split by COPD patients with versus without one or more diagnoses of other chronic respiratory diseases at baseline. (Patients with another chronic respiratory disease next to their COPD showed a higher hazard rate for the development of a first exacerbation (Cox hazard ratio 1.26; 1.17–1.36) compared to patients without another chronic respiratory disease). COPD: chronic obstructive pulmonary disease

Having another chronic respiratory disease beside COPD was also associated with risk of developing a first exacerbation (Cox hazard ratio 1 · 26; 1 · 17–1 · 36), see Fig. 2.

## Discussion

In this paper we explored the prevalence of comorbid chronic conditions and associations with exacerbation risk in a real-life cohort of primary care COPD patients. Our findings support the notion that comorbidities are rather rule than exception in patients with COPD [4], with 88% having at least one other chronic disease. Several comorbidities were associated with having frequent exacerbations, with heart failure, blindness/low vision and pulmonary cancer showing the strongest associations in terms of statistical significance. In contrast, diabetes was associated with a lower risk of having frequent exacerbations. Bronchiectasis/chronic bronchitis, heart failure and depression were the strongest predictors for developing a new exacerbation.

### Comparison with existing literature

Previous research has shown that cardiovascular, psychiatric, and metabolic comorbidity are highly prevalent in COPD patients, [8, 17] and our results confirm these findings. In addition to the finding by Rutten et al*.* [18] that unrecognized heart failure is rather common in elderly patients with stable COPD, our data also indicate that heart failure may increase the risk of having frequent exacerbations. Recent clinical trial data have shown correlations between several comorbidities and mortality risk if a COPD patient is admitted to hospital with an acute exacerbation [19, 20]. Our observations support the association between chronic comorbidity and exacerbation risk in a primary care study population, i.e., the COPD population without selection of any kind, which is unprecedented and impossible to derive from clinical trial populations [21].

We observed a trend towards statistical significance that COPD patients with dislipidemia had less frequent exacerbations compared to patients without dislipidemia (HR 0.92; *p* = 0.067). This observation seems to be in line with findings by Ingebrigtsen et al*.,* who recently reported that statin use for treatment of dislipidemia was associated with reduced odds of exacerbations in individuals with COPD [22] and findings by Chan et al. that hyperlipidemia in COPD was associated with decreased incidence of pneumonia and mortality in retrospective analyses of health insurance data [23]. Intuitively, the observed lower risk of frequent exacerbations in COPD patients with comorbid diabetes might be sought in GPs’ reluctance to prescribe oral corticosteroids in these patients because the impact this may have on glucose levels, but a survey among Dutch GPs showed that most of them do not adjust treatment of exacerbations to the presence of diabetic comorbidity [24]. Gastroesophageal reflux disease (OR = 1.25 (95% CI 1.03–1.50) in our analyses) was recognized as a significant predictor of acute exacerbations of COPD in a recent review by Lee et al [25]. A relationship between prostate disorders and exacerbations has not been described in the literature, but might be related to use of inhaled anticholinergics.

### Strengths and limitations

A strength of this study is the inclusion of >14 thousand COPD patients from a real-life, unbiased primary care setting. However, the main strength is not so much the uniqueness or even the size of our dataset. Other existing general practice databases essentially contain the same, or even more detailed data regarding diagnoses and medication prescriptions, [26–29] but the meticulousness with which we have looked at ALL chronic comorbidity, including recurrent episodes of conditions that are not necessarily chronic in all patients, seems unprecedented. Moreover, other existing databases with real-life general practice COPD data mainly stem from the UK and Denmark, and now there is also one available from the Netherlands. We intentionally applied minimal exclusion criteria in order to maximize generalizability of the results. Another strength is the wide range of chronic comorbidities investigated, summing up to a total of 82 conditions. Apart from all commonly known chronic comorbid diseases, we also included several recurrent diseases (i.e., depression, anxiety, anemia, dyspepsia, urinary tract infection) and applied criteria to define their chronicity based on disease specific guidelines (see Appendix 1). Inclusion of patients with recurrent diseases seems relevant when studying risk factors for COPD exacerbations, but has not been done in previous studies.

Our study was based on patients’ medical records in general practice. Limited agreement between medical record-based and objectively identified comorbidities of COPD [30] and undiagnosed comorbidity in COPD patients is common [18, 31]. This may have resulted in underestimation of the presence of comorbidity in our study population. The use of real-life data presents limitations, for instance the fact that patients’ smoking history and lung function could not be included because this information is not consistently and uniformly documented in general practice medical records. We chose to limit the analyses to comorbidities with a relatively high (i.e., ≥5%) prevalence. This may mean that comorbidities that are related to increased exacerbation risk but have a low prevalence rate in the COPD patient population were missed.

We defined an exacerbation as an oral corticosteroid prescription, which is the recommended treatment for acute exacerbations in Dutch COPD guidelines [15]. Consequently, mild exacerbations treated with bronchodilators only are not included in our analyses. Oral steroid prescriptions during GP out-of-office hours, emergency department visits and hospitalizations, and prescriptions by pulmonary specialists may not always have been included for all patients, as these are not automatically added to patients’ medical records in all electronic patient record systems. Because there is no international consensus about a definition that discriminates relapse of an earlier exacerbation from a new one, our (arbitrary) choice to use an interval of ≥14 days since the end date of the previous oral steroid prescription may have led to under- or overestimation of the number of exacerbations. Unfortunately, the rather crude prescription information did not allow us to look at the impact of comorbidities on the duration or progression of exacerbations. Although observational studies such as ours lack the rigorous internal validity that is typical for randomized controlled trials, they provide valuable insight into comorbidity prevalence in COPD and its relation with an important outcome, i.e., exacerbations. As such, our findings should be considered in conjunction with those arising from other study designs, including randomized trials.

### Clinical implications

Better knowledge about the role that comorbidity plays in COPD exacerbation risk may contribute to lower exacerbation rates in COPD patients through patient-tailored and systems medicine approaches. In turn, reduction of exacerbations may improve patients’ quality of life and prevent disability, hospitalizations, and mortality. A challenge for researchers is to find ways to enable physicians to take comorbidity into account when assessing COPD patients’ exacerbation risk. Putcha et al*.* developed a simple score that includes 14 comorbidities, where one point increase in comorbidity count was associated with 21% higher exacerbation risk [9]. However, their comorbidity score does not include comorbidities such as asthma, lung cancer and depression, while our results indicate that these comorbidities are also related to exacerbation risk. Neither does Putcha’s score take differences in exacerbation risk for different comorbidities into account. This highlights the importance of including a wide range of comorbid chronic conditions like we did in our study.

Beside Putcha’s comorbidity score, several prognostic indices to support COPD patient care have been developed, [32] most of them predicting prognosis in terms of mortality or hospitalization. Only few indices predict exacerbation risk and only one (the DOSE index [33]) has been developed and validated in primary care [34]. Comorbidity is not included in the existing prognostic indices, with the exception of the COTE index, which assesses mortality and not exacerbation risk [10, 11]. Our results may contribute to the development of a prognostic index that connects comorbidities with exacerbation risk to identify patients at highest risk, thereby potentially reducing disease progression.

## Conclusion

We have confirmed that many patients with COPD are affected by chronic comorbidities. Several highly prevalent as well as cardiopulmonary comorbidities appear to be independently associated with the risk of suffering from frequent exacerbations in our unbiased primary care patient population. Apart from clinical COPD guidelines advising that comorbidities should be diagnosed and treated appropriately, insight in patients’ comorbidity patterns could also be used to identify those that are more likely to suffer from frequent exacerbations. Further research is needed to assess opportunities of implementation of this knowledge in routine care, so that patient-centered COPD care that also takes comorbidity into account can become the standard. Ultimately this may contribute to reducing disease progression and reduce the significant burden that COPD and its exacerbations puts on patients and healthcare systems.

## Appendix 1

**Table 6:** List of 82 comorbidities included in comorbidity selection, sorted by prevalence (%) in the study population

Comorbiditiy	Prevalence (%)	Diagnosis	ICPC code	Inclusion criteria
Hypertension	35.2	Hypertension	K86, K87	ICPC code before 1-1-12
Coronary heart disease	19.2	Myocardial infarction/other ischemic heart disease	K75, K76, K76.02, K76.01	ICPC code before 1-1-12
		Angina Pectoris	K74, K74.01, K74.02	ICPC code before 1-1-12
Osteoarthritis	17.6	Artrose/spondylose wervelkolom	L84, L84.01, L84.02	ICPC code before 1-1-12
		Gonartrose	L90	ICPC code before 1-1-12
		Coxartrose	L89	ICPC code before 1-1-12
		Osteoarhritis, other	L91	ICPC code before 1-1-12
Diabetes	17.3	DM1, DM2	T90, T90.01, T90.02	ICPC code before 1-1-12
Peripheral vascular disease	14.3	Atherosclerose	K91	ICPC code before 1-1-12
		Intermittent claudication/Raynaud/Buerger	K92, K92.01, K92.02, K92.03	ICPC code before 1-1-12
		Other disease cardiovascular system	K99, K99.01, K99.02, K 99.03, K99.04, K99.05, K99.06	ICPC code before 1-1-12
Blindness & low vision	13.8	(Diabetic/hypertensive) retinopathy	F83, F83.01, F83.02	ICPC code before 1-1-12
		Maculadegeneratie	F84	ICPC code before 1-1-12
		Blindness/amblyopia	F94	ICPC code before 1-1-12
		Cataract	F92, F92.01	ICPC code before 1-1-12
Dyspepsia, Gastroesophageal reflux (GERD)	12.6	Stomach ulcer	D86.01	ICPC code before 1-1-12 AND (recode OR connection to episode) 12 months after first ICPC [35]
		Duodenal ulcer	D85	ICPC code before 1-1-12 AND (recode OR connection to episode) 12 months after first ICPC [35]
		Peptic ulcer, other	D86	ICPC code before 1-1-12 AND (recode OR connection to episode) 12 months after first ICPC [35]
		Oesophagus reflux with and without oesophagitis	D87, D87.01, D87.02, D84, D84.02, D84.03	ICPC code before 1-1-12 AND (recode OR connection to episode) 12 months after first ICPC [35]
Dislipidemia	11.5	Hypercholesterolemia/hypertriglyceridemia	T93, T93.01, T93.02, T93.03, T93.04	ICPC code before 1-1-12
Stroke & transient ischaemic attack	9.7	TIA (transient ischemic accident)	K89	ICPC code before 1-1-12
		CVA (cerebrovascular accident)	K90, K90.01, K90.02, K90.03	ICPC code before 1-1-12
Chronic kidney diease	9.5	Renal dysfunction	U99, U99.01	ICPC code before 1-1-12
Asthma	8.5	Asthma	R96, R96.01, R96.02	ICPC code before 1-1-2012 AND (recode OR connection to episode) 24 months after first ICPC code [36]
Hearing loss	8.1	Deafness	H84, H86, H85	ICPC code before 1-1-12
		Otosclerosis	H83	ICPC code before 1-1-12
Heart failure	7.9	(congestive) heart failure	K77, K77.01, K77.02	ICPC code before 1-1-12
		Pulmonary heart disease	K82	ICPC code before 1-1-12
Atrial fibrillation	7.5	Atrial fibrillation/flutter	K78	ICPC code before 1-1-12
Skin cancer	6.3	Skin cancer	S77.01, S77.02, S77.03, S77.04, S77	ICPC code before 1-1-12
Osteoporosis/osteopenia	6.3	Osteoporosis/osteopenie	L95, L95.01, L95.02	ICPC code before 1-1-12
Depression	5.6	Depressive disorder	P76, P76.01	ICPC code before 1-1-2012 AND (recode OR connection to episode) 24 months after first ICPC code [37, 38]
Thyroid disorder	5.6	Hypothyroidism	T86	ICPC code before 1-1-12
		Hyperthyroidism	T85	ICPC code before 1-1-12
Psoariasis	4.6	Psoriasis	S91	ICPC code before 1-1-12
Obesity	4.4	Adipositas	T82	ICPC code before 1-1-12
Anxiety	4.3	Somatoform disorder	P75	ICPC code before 1-1-2012 AND (recode OR connection to episode) 24 months after first ICPC code [39]
		Phobia	P79.01	ICPC code before 1-1-2012 AND (recode OR connection to episode) 24 months after first ICPC code [39]
		Anxiety disorder	P74, P 74.01, P74.02	ICPC code before 1-1-2012 AND (recode OR connection to episode) 24 months after first ICPC code [39]
		Obsessive - compulsive disorder	P79.02	ICPC code before 1-1-2012 AND (recode OR connection to episode) 24 months after first ICPC code [39]
		(chronic) functional somatic symtoms	P01, P78	ICPC code before 1-1-2012 AND (recode OR connection to episode) 24 months after first ICPC code [39]
		Post traumatic stress disorder	P02.01	ICPC code before 1-1-2012 AND (recode OR connection to episode) 24 months after first ICPC code [39]
Eczema	4.1	Atopic dermatitis	S87	ICPC code before 1-1-12
Heart valve disease	3.9	Heart valve disease	K83, K83.01, K83.02	ICPC code before 1-1-12
		Heart valve disease (rheumatic)	K71.02	ICPC code before 1-1-12
Diverticular disease of intestine	3.9	Colonic diverticula, diverticulitis	D92	ICPC code before 1-1-12
Alcohol problems	3.9	Chronic alcohol abuse	P15, P15.01, P15.02, P15.03, P15.04, P15.05, P15.06	ICPC code before 1-1-12
Rheumatoid arthritis, other inflammatory polyarthropathies & systemic connective tissue disorders	3.7	Rheumatoid arthritis/ankylosing spondylarthritis	L88.01, L88.02, L88	ICPC code before 1-1-12
Bronchiectasis/chronic bronchitis	2.8	Bronchiectasis/Chronic bronchitis	R91.02, R91, R91.01	ICPC code before 1-1-12
Irritable bowel syndrome	2.8	Irritable bowel syndrom	D93	ICPC code before 1-1-12
Venous insufficiency	2.4	Venous insufficiency	K99.04	ICPC code before 1-1-12
		Varicose ulcer	S97, S97.01	ICPC code AND (recode OR connection to episode) 3 months after first ICPC code [40]
Pulmonary cancer	2.4	lung/bronchial cancer	R84	ICPC code before 1-1-12
Recurrent urinary tract infection	2.3	Urinary tract infection, chronic/recurrent	U71, U71.01, U71.02	ICPC code AND (recode OR connection to episode) ≥3 times/year in 2011, 2012, 2013. Years start with 1e ICPC code. Minimal 8 weeks between each episode [41]
Breast cancer	2.3	Breat cancer	X76, X76.01	ICPC code before 1-1-12
Glaucoma	2.2	Glaucoma/verhoogde oogboldruk	F93, F93.01, F93.02, F93.03, F93.04	ICPC code before 1-1-12
Gout	2.0	Gout	T92	ICPC code AND (recode OR connection to episode) ≥3 times/year in 2011, 2012, 2013. Years start with 1e ICPC code. Minimal 22 days between each episode [42]
Prostate cancer	1.9	Prostate cancer	Y77	ICPC code before 1-1-12
Dementia	1.7	Alzheimer's disease/Senil dementia/Alzheimer/Multi-infarct dementia	P70.01, P70, P70.02	ICPC code before 1-1-12
Colorectal cancer	1.7	Colon cancer	D75	ICPC code before 1-1-12
		Rectal cancer	D75	ICPC code before 1-1-12
Epilepsy	1.4	Epilepsy	N88	ICPC code before 1-1-12
Bladder cancer	1.3	Bladder cancer	U76	ICPC code before 1-1-12
Sleep apnea syndrome	1.2	Sleep apnea syndrome	P0601	ICPC code before 1-1-12
Underfeeding/vitamine deficiency	1.2	Underfeeding/vitamine deficiency	T91, T05	ICPC code before 1-1-12 AND (recode OR connection to episode) 12 months after first ICPC
Inflammatory bowel disease	1.2	Crohn's disease/Ulcerative colitis	D94, D94.01, D94.02	ICPC code before 1-1-12
Personality disorder	1.2	Personality disorder	P80, P80.01, P80.02	ICPC code before 1-1-12
Prostate disorders	1.2	Prostatic hyperplasia/hypertrophy	Y85	ICPC code before 1-1-12
Other chronic pulmonary disease	1.1	Pulmonary tuberculosis	R70	ICPC code before 1-1-12
		Pneumoconiosis	R99.06	ICPC code before 1-1-12
		Sarcoidosis	R83.02	ICPC code before 1-1-12
Chronic liver disease	1	Cirrose/steatose	D97, D97.04, D97.05	ICPC code before 1-1-12
Genitourinary cancer, other	0.9	Genitourinary cancer, other	U75, U77, X77, Y78, Y78.01, Y78.03	ICPC code before 1-1-12
Blood(forming organs) and lymphatics disorder	0.8	Benign non specified neoplasm blood/lymphatic disorder	B75	ICPC code before 1-1-12 AND (recode OR connection to episode) 12 months after first ICPC [43]
		Haemophilia	B83.01	ICPC code before 1-1-12
		Congenital blood/lymphatic disorder	B79	ICPC code before 1-1-12
		Purpura/coagulation disorders/abnormal trombocytes	B83, B83.02, B83.06	ICPC code before 1-1-12
Schrizophrenia/non-organic psychosis/bipolar disorder	0.8	Schizophrenia	P72	ICPC code before 1-1-12
		Psychosis non specified	P98	ICPC code before 1-1-12
		Bipolar	P73.02	ICPC code before 1-1-12
Migraine	0.8	Migraine	N89	ICPC code before 1-1-12 AND (recode OR connection to episode) 12 months after first ICPC [44]
Cancer oropharynx, oesophageal, stomach	0.8	Cancer of the mouth/pharynx	D77.02, D77.03	ICPC code before 1-1-12
		Oesophageal cancer	D77.01, D77	ICPC code before 1-1-12
		Cancer of stomach	D74	ICPC code before 1-1-12
Other psychoactive substance misuse	0.7	Substance abuse	P19, P19.01, P19.02	ICPC code before 1-1-12
Parkinson's disease	0.6	Parkinson's disease	N87.01, N87	ICPC code before 1-1-12
Other chronic skin disease/neoplasm (sub)cutis	0.6	Neoplasm cutis, subcutis non specified	S80, S80.01, S81, S83, S83.01, S83.02	ICPC code before 1-1-12
		Vitiligo/lichen planus	S99.04, S99.06	ICPC code before 1-1-12
Viral hepatitis	0.6	Hepatitis B	D72.02, D72.04	ICPC code before 1-1-12
		Hepatitis C	D72.03, D72.05	ICPC code before 1-1-12
		Hepatitis	D72	ICPC code before 1-1-12
Uterine cervical cancer	0.5	Uterine cervical cancer	X75	ICPC code before 1-1-12
Learning disability’/Mental retardation	0.4	Mental retardation	P85	ICPC code before 1-1-12
		Specified learning problems	P24. P24.01, P24.02, P24.03	ICPC code before 1-1-12
Laryngeal/throat cancer	0.4	Laryngeal/troat cancer	R85	ICPC code before 1-1-12
Hodgkin disease	0.4	Hodgkin disease	B72, B72.01, B72.02	ICPC code before 1-1-12
Carcinoma, other	0.4	Carcinoma, other	D77.04, T71, W72, L71, L71.01	ICPC code before 1-1-12
Chronic sinusitis	0.3	Chronic sinusitis	R75.02	ICPC code before 1-1-12
		Acute Sinusitis	R75.01 en R75	ICPC code AND (recode OR connection to episode) ≥3×/year in 2011, 2012, 2013. Years start with 1e ICPC code. Minimal 29 days between each episode. [45]
Glomerulonephritis/nephrosis	0.3	Glomerulonephritis	U88	ICPC code before 1-1-12
Congenital cardiovascular anomaly	0.2	Congenital cardiovascular anomaly	K73, K73.01, K73.02	ICPC code before 1-1-12
Leukaemia	0.2	Leukaemia	B73	ICPC code before 1-1-12
Lymphoma/multiple myeloma/other blood cancer	0.2	Lymphoma/multiple myeloma/other blood cancer	B74.01, B74	ICPC code before 1-1-12
Anaemia	0.1	Pernicous/folic acid anaemia	B81, B81.01, B81.02	ICPC code before 1-1-12 AND (recode OR connection to episode) 12 months after first ICPC [43]
		Haemolytic anaemia	B78, B78.01, B78.02, B78.03	ICPC code before 1-1-12
Anorextia or bulimia	0.1	Anorexia nervosa	T06, T06.01, T06.02	ICPC code before 1-1-12
Coeliakie	0.1	Coeliakie	D99.06	ICPC code before 1-1-12
Endometrial cancer	0.1	Endometrial cancer	X77.01	ICPC code before 1-1-12
Metastases; unknown origin	0.1	Metastases; unknown origin	A79	ICPC code before 1-1-12
Multiple sclerosis	0.1	MS (multiple sclerosis)	N86	ICPC code before 1-1-12
Ovarian cancer	0.1	Ovarian cancer	X77.02	ICPC code before 1-1-12
Pancreatic cancer	0.1	Pancreatic cancer	D76	ICPC code before 1-1-12
Testis cancer	0.1	Testis cancer	Y78.02	ICPC code before 1-1-12
Brain cancer (recall: Nervous system cancer)	0	Brain cancer (recall: Nervous system cancer)	N74	ICPC code before 1-1-12
HIV/AIDS	0	HIV; AIDS	B90, B90.01, B90.02	ICPC code before 1-1-12

## Appendix 2

**Table 7:** List of comorbidity categories

Categories of chronic disease	Disease
Cardiovascular	Hypertension
	Coronary heart disease
	Congenital cardiovascular anomaly
	Heart failure
	Stroke & transient ischaemic attack
	Atrial fibrillation
	Heart valve disease
	Venous insufficiency
	Peripheral vascular disease
Respiratory	COPD
	Asthma
	Sleep apnea syndrome
	Chronic sinusitis
	Other chronic pulmonary disease
	Bronchiectasis/chronic bronchitis
Mental Health	Depression
	Anxiety disorder
	Alcohol problems
	Other psychoactive substance misuse
	Schrizophrenia/non-organic psychosis/bipolar disorder
	Anorextia or bulimia
	Personality disorder
	Learning disability’/Mental retardation
Musculoskeletal	Rheumatoid arthritis, other inflammatory polyarthropathies & systemic connective tissue disorders
	Gout
	Osteoporosis/osteopenie
	Osteoarthritis
Eye and Ear	Hearing loss
	Glaucoma
	Blindness & low vision
Urogenital (Male and female)	Chronic kidney diease
	Glomerulonephritis/nephrosis
	Recurrent urinary tract infection
	Prostate disorders
Skin	Eczema
	Psoriasis
	Other chronic skin disease/neoplasm (sub)cutis
Digestive	Diverticular disease of intestine
	Dyspepsia, Gastroesophageal reflux
	Irritable bowel syndrom
	Inflammatory bowel disease
	Coeliakie
	Chronic liver disease
Endocrine, metabolic and nutrition	Underfeeding/vitamine deficiency
	Diabetes
	Dislipidemia
	Obesity
	Thyroid disorder
Neurological	Dementia
	Epilepsy
	Migraine
	Parkinson's disease
	Multiple sclerosis
Blood(forming organs) and Lymphatics	Anaemia
	Blood (forming organs) and lymphatics disorder
Infectious	Viral hepatitis
	HIV/AIDS
Non-pulmonary cancer	Testis Cancer
	Cancer oropharynx, oesophageal, stomach
	Cancer Colorectal
	Pancreatic cancer
	Laryngeal/troat cancer
	Breast cancer
	Ovarian cancer
	Endometrial cancer
	Uterine cervical cancer
	Prostate cancer
	Bladder cancer
	Genitourinary cancer, other
	Brain cancer (recall: Nervous system cancer)
	Hodgkin disease
	Leukaemia
	Lymphoma/multiple myeloma/other blood cancer
	Metastases; unknown origin
	Carcinoma, other
	Skin cancer
Pulmonary cancer	Pulmonary cancer

## Appendix 3

**Table 8:** Baseline characteristics of the initial population of all COPD patients, the patients who were lost to follow-up, and the patients with full follow-up

	All COPD patients (*n*=16,427)	Patients lost to follow-up (*n*= 1,824)	Patients with full follow-up (study population)^a^ (*n*=14,603)
Patient characteristics			
Sex, male, n (%)	8,682 (52·9)	933 (51·2)	7,749 (53·1)
Age at study baseline, years; mean (SD; range)	66·9 (11·6; 40–111)	70·1 (12·0; 40–111)	66·5 (11·5; 40–110)^‡^
Full dataset available (censored data), n (%)			
Full data available	14,603 (88·7)		
Deceased	541 (3·0)	541 (29·7)	N/A
Moved	223 (1·3)	223 (12·2)	N/A
Nursing home	36 (0·2)	36 (2·0)	N/A
Unknown	1024 (6·2)	1024 (56·1)	N/A
Comorbidity data			
Number of comorbid diseases^b^, mean (SD; range)	3·0 (2·3;0–20)	3·4 (2·5; 0–16)	3·0 (2·3;0–20)^‡^
Number of comorbid diseases categories^b^, n (%)			
0	1,951 (11·9)	174 (9·5)	1,777 (12·2)
1 or 2	5,891 (35·9)	586 (32·1)	5,305 (36·6)
3 or 4	4,797 (29·2)	539 (29·6)	4,258 (29·2)
5 and more	3,788 (23·1)	525 (28·8)	3,263 (22·3)^‡^
Exacerbations data			
Number of exacerbations, mean (SD; range)	0·72 (1·5;0–15)^c^	0·46 (1·0;0–11)^c^	0·75 (1·5;0–15)

*SD* standard deviation, *N/A* not applicable

^*^ *p*<0.05, ^†^ *p*<0.01, ^‡^ *p*<0.001

^a^ *p*-values displayed are calculated for the difference between patients lost to follow-up *versus* patients with full follow-up. Chi-square tests for categorized variables and independent t-tests for continuous variables. *p*<0·05 was considered statistically significant

^b^Presence of any type of comorbid disease was assessed at study baseline, i.e. 1 January 2012

^c^Mean number of exacerbations during the study period, 1 January 2012 – 31 December 2013. For the columns ‘all COPD patients’ and ‘Patients lost to follow-up’ these rates cannot be converted into annual rates because of incomplete observation time in the patients who were lost to follow-up

Baseline characteristics of the study population grouped by low (<2/year) *versus* high (≥2/year) exacerbation rate are reported in Table 1

## Abbreviations

ATC: Anatomical therapeutic chemical
CKD: Chronic kidney disease
COPD: Chronic obstructive pulmonary disease
GERD: Gastroesophageal reflux disease
GP: General practitioner
ICPC: International classification of primary care
N/A: Not applicable
OR: Odds ratio
SD: Standard deviation
TIA: Transient ischemic attack
UK: United Kingdom
US: United States
